# Mothers as Home DJs: Recorded Music and Young Children’s Well-Being During the COVID-19 Pandemic

**DOI:** 10.3389/fpsyg.2021.637569

**Published:** 2021-05-06

**Authors:** Eun Cho, Beatriz Senoi Ilari

**Affiliations:** ^1^Experimental Acoustics Research Studio, University of California, Riverside, Riverside, CA, United States; ^2^Music Teaching and Learning, Thornton School of Music, University of Southern California, Los Angeles, CA, United States

**Keywords:** music listening, parenting during COVID-19, young children, home musical environment, mood, emotions

## Abstract

As the COVID-19 pandemic continues to disrupt our lives in unimagined ways, families are reinventing daily rituals, and this is likely true for musical rituals. This study explored how parents with young children used recorded music in their everyday lives during the pandemic. Mothers (*N* = 19) of child(ren) aged 18 months to 5 years living in the United States played the role of home DJ over a period of one week by strategically crafting the sonic home environment, based on resources provided by the authors, in response to their children’s mood and state. Using a newly developed data collection tool, inspired by the Experience Sampling Method, a total of 197 episodes were collected about children’s engagement with recorded music. Findings showed that while mothers utilized music to fulfill various emotional needs, they tended to use it to maintain or reinforce their child’s positive mood rather than to improve a negative mood. Also, mothers’ reports suggested various ways that young children engaged with music, being aligned with the multimodal nature of their musical experiences. Lastly, mothers reported that their strategic approaches to use recorded music seemed to help their children feel less distressed and happier, and this, in turn, aided in the reduction of some of the burdens associated with parenting.

## Introduction

The COVID-19 pandemic has disrupted our lives in unimagined ways. Job losses, illness and social distancing have generated a great amount of stress in adults and children alike. The closure of schools allied with the need to homeschool children, the shift to remote working, the lack of assistance with childcare and housework, and of course, fears of becoming ill or having someone ill in the family have added time pressure, anxiety, vulnerability, stress and burnout in parents from across the globe (Abdellatif and Gatto, 2020; American Psychological Association [APS], 2020; Brown et al., 2020; Hiraoka and Tomoda, 2020; Ilari et al., under review; Spinelli et al., 2020). Families with young children (aged 0 to 5 years) have been in a particularly vulnerable position due to the multiple demands associated with childcare during the early years of life which are incongruent with the disruptions associated with the pandemic (Davenport et al., 2020). The Center for Translational Neuroscience (2020) surveyed 1000 American families with children under the age of 5 weekly starting in April 2020, and found 63% of parents to report a lack of emotional support since the beginning of the pandemic. Emotional support—from family, friends and communities—is known to be a protective factor against the negative effects of stress (Center for Translational Neuroscience, 2020).

Elevated levels of stress are a serious concern for individuals and communities alike. Prolonged exposure to stress has a toll on the developing body and brain, and may negatively impact learning, memory and the ability to regulate responses to stress (National Scientific Council on the Developing Child, 2014). The quality of child-caregiver interactions is known to play critical roles in stress regulation in childhood. This is understandable, as “stressors that impede one family member may lead to change in the functioning of all family members” (Prime et al., 2020). In recent investigations, stress was found to be a mediating factor between parental perceptions of the impact of COVID-19 and parent-child closeness (Chung et al., 2020), effects of quarantine and children’s well-being (Spinelli et al., 2020), and perceptions of the pandemic and harsh parenting (Chung et al., in press). These studies not only confirm the toll of COVID-19 on human mental health and well-being, but also reinforce the need to improve the lives of children and families by countering stress during these difficult times.

Musical enjoyment—through listening, moving, or playing—has been shown to induce positive emotions and reduce stress (see Yehuda, 2011). Music is, in fact, one of the oldest forms of stress reduction (Croom, 2012). The associations between musical engagement and well-being have been documented (see Croom, 2012). In therapeutic settings, music has been used to promote well-being in infants, children and adults (Croom, 2012), and more recently, in essential health workers during the COVID-19 pandemic (Giordano et al., 2020). Listening to music can elicit intensely pleasurable experiences in adults and may lead to the release of dopamine—popularly known as “the feel-good neurotransmitter.” (Salimpoor et al., 2011). As Heggli et al. (2019) suggested, “the enjoyment of music is a uniquely human capability and perhaps one of the most important pleasures in life” (p. 205). Recent studies suggest that music has been used to regulate emotions and to counter sadness and despair since the start of the pandemic (Corvo and DeCaro, 2020). This is consistent with earlier research suggesting various reasons for music listening, including mood regulation, relaxation, enjoyment, safe time passing, masking external noises and expressing social relatedness (Randall and Rickard, 2017).

Parents are known to listen to music with young children for similar reasons, oftentimes with expectations that music will positively impact their youngsters’ lives (Lamont, 2008). Music has been used to regulate mood and behaviors of infants, young children and their caregivers (Trehub and Schellenberg, 1995; Shenfield et al., 2003; Trehub et al., 2010). But unlike some adults who often sit and listen quietly to a musical piece, young children are known to listen to music in idiosyncratic ways. Their listening modes are directly linked to levels of intention in learning in the early years (see Young and Ilari, 2018; Ilari, in press). Young children’s listening may be *implicit* in situations where there is music in the background but they are not responding overtly to it; *reactive* when children respond overtly to music by moving, singing along, or extending it in some way; *deliberate* when there is an explicit learning intention on the part of the adult or listening companion as in an education program, and *self-initiated* when the child plays a more agentic role and initiates (and also terminates) his or her own listening experiences (Ilari, in press). Young children’s music listening is also of a multimodal nature. As Gardner (1980) explained many years ago, “the child sings as he draws, dances as he sings, tells stories while at play in the bathtub or in the backyard. Children move readily and even eagerly from one form to another, combine the forms, and play them off one against another” (p. 99). Digital technologies have afforded children with additional forms of music listening, oftentimes requiring that they engage more than just the hearing sense, like when a child “listens” to a musical video on a screen (Young and Wu, 2019). A recent example is that of “Baby Shark,” a Korean pop musical video published on YouTube that went viral worldwide, being one of the few children’s songs to ever hit the Billboard (Basu, 2019). The case of “Baby Shark” also illustrates how young children’s musical experiences are associated with their own musical preferences, choices and agency, as described in earlier works (see Lamont, 2008; Custodero et al., 2016).

Although young children move spontaneously, vocalize and sing along in response to recorded music, they are not always able to initiate such listening experiences, particularly when the latter is predicated on access to specific audio devices. Oftentimes, it is the parent or older companion who provides music listening experiences for young children, particularly during babyhood and toddlerhood. That is, parents and caregivers shape the sonic environment in the home by curating children’s musical experiences from very early on (Ilari, 2018). Parents curate musical experiences in the home based on their own previous musical experiences and taste (Custodero and Johnson-Green, 2003), song characteristics (Sulkin and Brodsky, 2015), and children’s mood and time in the routine (Trehub and Schellenberg, 1995). Parents are also known to craft and adjust musical experiences in the home, based on family routines and children’s reactions to the different songs and musical pieces that they encounter (Ilari, 2018). Over time, shared musical experiences can turn into family musical rituals (Ilari, 2018), leaving imprints that children may carry with them for the rest of their lives (Boer and Abubakar, 2014). In times of COVID-19, parents, families and communities are reinventing rituals (Imber-Black, 2020), and this is probably true for family musical rituals. We speculate that parents, who are keenly aware of and respond to children’s moods and behaviors by purposefully crafting the sonic environment in the home, may help children feel less distressed and happier. Such positive feelings, in turn, may also aid in the reduction of parental anxieties and stress during the period of confinement associated with the COVID-19 pandemic.

This exploratory study sought to explore how parents with young children use recorded music in their everyday lives during the pandemic, using a data collection tool that we developed based on the Experience Sampling Method or ESM (Larson and Csikszentmihalyi, 2014). The first aim was to examine parents’ experiences of utilizing recorded music for their children in the home environment over the course of one week called “Parents as Home DJs week.” Three specific research questions guided our work: (1) in what situation do parents use music?; (2) what kinds of music do they select for their children in the given situation?; and (3) how do parents perceive the effects of music listening, particularly in relation to their overall well-being and that of their children? We also aimed to test a modified version of ESM that we designed specifically for this study to collect data on young children’s musical experiences under the unique challenges of the COVID-19 pandemic.

## Materials and Methods

### Participants

The population of interest for this study was primary caregivers of young children, aged 18 months to 5 years, living in the United States. While a total of 19 participants voluntarily took part in the study, all of them were mothers, which is consistent with many parenting studies (e.g., Williamson et al., 2017; Mingebach et al., 2018). Most mothers were between 30 and 39 years of age. In terms of occupation, while six participants were stay-at-home-mothers (32%), others had varying types of jobs, including arts-related (26%), education-related (21%), and health-related (5%) ones. Participating mothers tended to be highly educated, as about a half of them (53%) held at least one graduate degree. Seven held bachelor’s degrees (37%), and two mothers had high school as their highest education level (10%). In terms of formal music training experiences, two mothers had no prior music training experience (11%) while six responded having some kinds of music lessons in K-12 school years (32%) and another five having music lessons in the school years with additional engagements in various community music programs (26%). The remaining six participants held a degree in music (31%). While nine mothers had more than two children, seven had two children under the age of 5 and the other two reported having at least one child older than 5 years of age in addition to their younger child. In terms of ethnicity, although this study was open to any primary caregivers of young children living in the United States, all participants identified themselves as Asian-American.

### Data Collection Tools

The outbreak of COVID-19 limited human contact, which forced researchers to make use of creative tools for data collection, most of which involving digital technologies (e.g., Rogers et al., 2020). As we aimed to collect data on parents’ experiences of being a home DJ in daily life during the pandemic, we devised a modified version of the ESM, based on the methodology used by Lamont (2008). The ESM, a structural diary technique to gather systematic self-reports of subjective experiences in the participants’ natural environment (Larson and Csikszentmihalyi, 2014), has been incorporated in a wide range of fields and in varying ways. Lamont (2008) used mobile telephones to collect data on children’s everyday musical engagement by making calls to parents up to three times daily over a period of one week. Prior to the actual study, she also made a personal visit to each participating family to explain the study (Lamont, 2008).

Inspired by Lamont’s design, we developed a data collection tool that enabled us to communicate with participants and collect data without face-to-face contact by incorporating three types of digital technologies: social media platforms (i.e., Instagram and Facebook), an online video platform (i.e., YouTube), and an online survey tool (i.e., Qualtrics). Instagram (IG) served as a main platform for this study with four distinct functions: (1) to give instructions about participation, (2) to encourage active participation during the study week, (3) to give access to resources, and (4) to communicate with the participants. Since the study required participants to complete multiple tasks over a period of time, we posted various infographics to provide detailed instructions on the study and how to participate. We also posted musical parenting resources regularly to help participants become aware of the significance of music in their children’s lives, and to encourage their active roles as a home DJ. Participants were able to access music playlists (see below) and daily surveys through IG. Finally, daily messages were sent to individual participants via Direct Message (DM) to remind them about the daily survey and to keep the communication channel open, in case there were any questions. Overall and given the challenging circumstances with the pandemic, IG was an effective tool to sample participants’ experiences in the home environment, allowing us to collect participants’ daily reports over the course of a week while having close and convenient access to participants, yet without face-to-face interactions.

### Musical Stimuli: The Playlists

Capitalizing on the multimodality of music listening in early childhood (see Marsh, 2010; Kumpulainen and Gillen, 2019; Ilari, in press; Lewis, 2020), the study authors, who are active music educators with extensive experiences in early childhood music, created nine music playlists using YouTube. Several criteria were considered when selecting music for the playlists: (1) enjoyment - music that could be enjoyed by both young children and their adult caregivers; (2) diversity of genres and styles - music from a variety of genres/styles (e.g., folk songs, pop, children’s songs, jazz, instrumental music); (3) cultural diversity - music from different parts of the world (e.g., Brazil, Korea, Senegal, France); and (4) preferably music that was not based on electronic/digital processes. We collected about 100 pieces of music and undertook a thorough examination of their contents, audio and recording quality. We then curated each playlist, consisting of 10–12 pieces of music, around a unique theme (as suggested by their titles) to carefully match intended arousal levels and mood. Each playlist was linked with specific moods (e.g., lively, calm, upset) to reinforce their potential use within the child’s routines. Table 1 depicts the contents of the playlists with examples of titles of pieces and songs.

**TABLE 1:** Descriptions of the playlists.

**Playlist title**	**Mood vocabulary**	**Examples**
*(1) Always Lively Vibes*	Lively, playful, interested	“Here Comes the Sun” (The Beatles) “Les Cucurbitacés” (Claude Bolling) from France
*(2) Want to Soothe Fussy Child?*	Upset, frustrated, distressed	“What a Wonderful World” (Louis Armstrong) “Catch the Moon” (Lisa Loeb)
*(3) Feel Like Dancing?*	Cheerful, happy, excited	“I Hope I Get It” (from Chorus Line) “Happy” (Pharrell Williams)
*(4) Everyday Inspiration*	Interested, focused, peaceful	Mozart Violin Concerto No. 5 3^*rd*^ mvmt. “Come Fly with Me” (Beegie Adair)
*(5) Good Morning, Rise and Shine*	Lively, playful, interested	“Fatou Yo” (Touré Kunda) from Senegal “Bicicleta” (Palavra Cantada) from Brazil
*(6) Peaceful Minds*	Calm, peaceful, drowsy	Debussy “Clair de Lune” “Tanti Anni Prima” (Ara Malikian)
*(7) Playtime Yay!*	Excited, energized, lively	“Banana Cha-cha” (Momoland) from South Korea “El Soldado Trifaldón” (Tikitiklip) from Chile
*(8) Toddlers’ Favorites!*	Cheerful, lively, happy	“My Happy Song” (Super Simple Song)
*(9) Keep Calm and Relax*	Excited, energized, lively	“Olélé Moliba Makasi” (Jean-Marie Bolan hassa) from Congo “Makun” (Sylla Mama) from Mali

### Procedure

This study was approved by the Institutional Review Board (IRB) of the University of Southern California. Ten days prior to the Parents as Home DJs week, we posted online flyers on multiple parent groups on Facebook. To take part in the study, parents were invited to fill out an online survey, which consisted of three parts: (1) parent’s demographic information, including measures for psychological well-being and perceived satisfaction with their parental role; (2) child information; and (3) information about their daily life with their children. Upon the completion of the initial survey, participants were guided to follow the study’s IG account. They received a welcoming message via DM and were asked to regularly check out the daily posts on IG. Examples of daily posts included detailed instruction for the Parents as Home DJs week, musical parenting resources, and introductions to each playlist. Access to the playlists was also provided to participants, who were invited to explore and familiarize themselves with the music. A day before the Parents as Home DJs week, we communicated with each participant via DM to make sure that they fully understood the instructions and to answer any questions.

The Parents as Home DJs week took place on the fourth week of August in 2020. Parents’ role as a Home DJ was described as following:

*The real role of a DJ is, at first, selection—picking music that is right for the moment—and also having a sense of when to play it. What differentiates a good DJ from a not-so-great DJ is knowing how to adapt to the audience (in our case, YOUR CHILD!).*

During the study week, participants carefully observed their children’s mood throughout the day, looking for two specific moments to use music: (1) when the child was in an uncomfortable state that they wanted to help the child feel better; and (2) when the child was in a positive mood that they wanted to maintain the mood longer. When participants detected any of these situations, they were instructed to select and play music from one of the playlists, possibly considering their themes, or to use any other music of their choice. Participants then watched out for any effect the music had on their child. Participants tried this DJ role at least three times a day and reported their experiences on a daily survey each day. The daily survey required participants to report (1) the selected playlist; in case they used other repertoires, they were asked to offer specific information about the music; (2) the specific situation in which they decided to put on the music; (3) their child’s mood and emotional states at the moment; (4) any noticeable effect of the music on their child; and (5) an optional open-ended comment for the episode. Participants could report up to three episodes each day. The daily survey was available anytime between 9AM and midnight each day. Participants were also invited to share any photos and videos of the moment if they wished. At the end of the week, participants also answered an exit question to offer their impressions of DJ-ing for their children.

### Data Analysis

Data from the initial and daily surveys were analyzed separately. For the initial survey, data were analyzed descriptively. Participating mothers’ ratings on their psychological well-being (3 items) and satisfaction with their parental role (2 items) were averaged, with each final score ranging from 0 to 10. Independent-sample t-tests and one-way ANOVAs were performed to determine if differences existed on mothers’ psychological well-being and satisfaction with their parental role due to their involvement in childcare, number of children, occupation (stay-at-home-mother Y/N), and the frequency of music use with children. Assumptions of normality and homogeneity of variance were met.

During the Parents as Home DJs week, a total of 197 episodes were collected from the daily survey from all 19 mothers. In the daily survey, there were seven options to choose to indicate the situation mothers decided to put on music for their children. These were re-arranged based on children’s reported mood (positive vs. negative) and activity level (from high to low) at the time (see Table 2). The nine playlists were grouped into 4 categories with Level 1 being the most arousing and stimulative and Level 4 being the most relaxing and sedative. Out of 197 episodes, 19 were related to music from participants’ own repertoire. This repertoire was analyzed based on its arousal levels and classified into one of the four categories. A total of 10 media files (5 photos and 5 videos) were submitted by mothers to supplement written reports on individual episodes; however, we did not include them in our final analysis due to a lack of information of specific context.

**TABLE 2:** Situations in which mothers used recorded music.

**Mood**	**Activity level**	**Situation**			**Child’s state**		
			***N***	**%**		***n***	**%**
*Positive*	*Higher*	My child was hyperactive	19	9.6	Playful	14	28.0
	∣∣				Happy	10	20.0
	∣∣				Excited	10	20.0
	∣∣	My child was happy/cheerful/lively	70	35.5	Playful	53	35.6
	∣∣				Happy	51	34.2
	∣∣				Interested	17	11.4
	∣∣	My child was focusing on him/herself	25	12.7	Focused	19	32.8
	∣∣				Happy	12	20.7
	∣∣				Interested	12	20.7
	∣∣	My child was rested/relaxed	31	15.7	Calm	24	57.1
	∣∣				Happy	5	11.9
	*Lower*				Drowsy	4	9.5
*Negative*	*Higher*	My child was sad/upset/frightened	4	2.0	Uncomfortable	2	25.0
	∣∣				Upset	1	12.5
	∣∣				Sad	1	12.5
	∣∣	My child was fussy/cranky/irritable	22	11.2	Distressed	11	22.0
	∣∣				Upset	10	20.0
	∣∣				Annoyed	9	18.0
	∣∣	My child was tired/sleepy/hungry	26	13.2	Calm	14	37.8
	∣∣				Drowsy	8	21.6
	*Lower*				Annoyed	4	10.8

## Findings

Findings from the initial and daily surveys are reported separately. We begin with a discussion of study findings from the initial survey: COVID-19, parenting and mothers’ use of music with children prior to participation in the Parents as Home DJs week. Next, findings from the daily survey during the Parents as Home DJs week are presented, specifically in light of three main aspects: Situations, repertoire selection, and modulating music to promote well-being.

### COVID-19 and Parenting

As noted, all participants in this study were mothers and most (90%) reported being responsible for over 75% of childcare daily. More than half of the mothers (53%) reported that their involvement in parenting had increased since the outbreak of COVID-19. Mothers engaged in a variety of activities with their children in daily life, with reading, playing toys/games, and arts/crafts being the most popular (84%), followed by music (74%) and screen time (74%). Other activities, such as sports (68%), house chores (47%), cooking (37%), academics (37%) and religious activities (37%), were also incorporated into children’s routines. Mean scores of mothers’ perceived psychological well-being and satisfaction with their parental role are presented in Table 3. An independent-samples t-test and one-way ANOVAs were performed to determine if mothers’ perceived psychological well-being and satisfaction with their parental role were different due to their involvement in childcare, number of children, and occupation (stay-at-home-mother Y/N); however, no significant differences were found.

**TABLE 3:** Descriptive data on mothers’ psychological well-being and satisfaction.

	**Psychological**		**Satisfaction with**	
	**well-being**		**parental role**	
	***M***	**SD**	***M***	**SD**
***Overall* (*N* = 21)**	**6.98**	**1.77**	**7.00**	**1.96**
*Parental Involvement*				
100% (*n* = 11)	6.79	0.63	6.95	0.68
75% (*n* = 7)	7.62	0.57	7.36	0.70
50% (*n* = 3)	6.22	0.22	6.33	0.60
*# of Children*				
1 Child (*n* = 10)	6.97	0.65	6.80	0.78
2 + Children (*n* = 11)	7.00	0.47	7.18	0.44
*Stay-at-home mother*				
Yes (*n* = 6)	6.67	0.97	6.67	1.24
No (*n* = 15)	7.11	0.39	7.13	0.38

### Mothers’ Uses of Music With Children

Initial survey data indicated that all mothers used recorded music for their children in daily life although the frequency varied across households. Almost 60% of the mothers reported playing recorded music often; 32% did it occasionally, and only two mothers (9%) reported that they rarely played recorded music for their children. Functions of and reasons for music listening varied, with dancing with children being the most popular (79%). Many mothers reported using music to entertain and get their children “pumped up” (68%), and to reduce boredom (53%). Music was also used to help children get creative (47%), to maintain or improve mood (42%), and to create an atmosphere at home (37%). Mothers’ choice of repertoire for children were equally varied. Only a handful of mothers reported sharing their own musical tastes with their children. For example, although many mothers enjoyed pop/rock/hip-hop (63%), jazz (58%), and R&B/Soul (37%), less than half of them reported sharing these styles/genres with their children. Two exceptions were classical and religious music. More than two-thirds of mothers, who reported liking classical and/or religious music, reported playing these genres for their children. Meanwhile, children’s music (95%) and soundtracks from Children’s TV shows and Disney movies (63%) were included in the listening repertoire of most families, with six mothers reporting exclusively playing children’s music for their children. When asked to offer examples of their child’s favorite listening repertoires, songs that involve multi-modal listening were often reported (e.g., Pinkfong, Cocomelon, Super Simple Song, Sesame Street)—ones typically accessed through online music/video streaming services such as YouTube and Netflix.

In addition, data from the initial survey suggested potential effects of using recorded music at home on the well-being of mothers. A one-way ANOVA indicated that mothers’ psychological well-being, *F*(3,17) = 3.37, *p* = 0.043, $\eta_{p}^{2}$ = 0.373, and satisfaction with their parental role, *F*(3,17) = 3.51, *p* = 0.038, $\eta_{p}^{2}$ = 0.382, differed as a function of frequency of music use with their children. Tukey *post hoc* analysis revealed that the psychological well-being of mothers who used music often with their children (*M* = 8.14, *SD* = 1.19) was significantly higher than that of mothers who rarely listened to music (*M* = 4.67, *SD* = 1.86). Also, mothers who used music often (*M* = 8.50, *SD* = 1.32) were more satisfied with their parental role than mothers who only used music occasionally with their children (*M* = 5.79, *SD* = 2.12).

### Mothers as Home DJs

During the Parents as Home DJs week, participating mothers played the role of home DJ and reported their experiences by filling out a daily survey. While the survey was available from 9AM to midnight each day, mothers mostly filled them out at night as 70% of 197 episodes were recorded after 9PM. This suggests that mothers’ reports tended to be retrospective. The mean duration that mothers spent to fill out the survey was 4.9 min (*SD* = 5.4 min). Considering that the survey consisted of 4 multiple-choice questions and an optional open-ended question for each episode (they were allowed to report up to 3 episodes daily), we can assume that participants’ responses were deliberate.

#### Situations and Repertoire Selection

Table 4 summarizes mothers’ repertoire selections in different situations. Mothers reported using music most often when children were in a positive mood and exhibited a relatively high activity level (89 episodes). When children were happy/cheerful/lively (70 episodes), arousing and stimulative music, Level 1 (48.6%) and Level 2 (38.6%), were frequently selected. However, in moments when children were even more agitated and excited (19 episodes), maternal musical choices varied according to individual situations. For example, more arousing and stimulative music, Level 1 (26.3%) and Level 2 (31.6%), were chosen when mothers wanted children to stay active and energetic. On the other hand, when mothers wanted to calm down overactive children, they selected sedative and calming music (31.6%). Music was also used when children were in a positive mood but exhibited a relatively low activity level (i.e., when the child was rested/relaxed or self-focused; 56 episodes). Although mothers’ overall goal was to maintain their child’s positive mood, their music selection appeared to vary according to desired effects. When they wanted to keep their children focused and relaxed for a longer time, they were more likely to select Level 2 (28.6%) or Level 3 (30.4%). Level 4, in turn, was chosen (26.8%) particularly when mothers wanted their children to chill out and ultimately fall asleep. Music was also used to modulate children’s mood in situations where the child was fussy/cranky/irritable, sad/upset/frightened, or tired/sleepy/hungry (52 episodes). In those episodes, relatively low arousing and sedative music, Level 3 (46.2%) and Level 4 (28.8%), were commonly selected.

**TABLE 4:** Situations and mothers’ repertoire selection.

**Mood**	**Activity level**	**Situation**		**Music selection**		
			***n***		***n***	**%**
*Positive*	*Higher*	My child was hyperactive	19	Level 1	5	26.3
	∣∣			Level 2	6	31.6
	∣∣			Level 3	2	10.5
	∣∣			Level 4	6	31.6
	∣∣	My child was happy/cheerful/lively	70	Level 1	34	48.6
	∣∣			Level 2	27	38.6
	∣∣			Level 3	1	1.4
	∣∣			Level 4	8	11.4
	∣∣	My child was focusing on him/herself &	56	Level 1	8	14.3
	∣∣	My child was rested/relaxed		Level 2	16	28.6
	∣∣			Level 3	17	30.4
	∣∣					
	*Lower*			Level 4	15	26.8
*Negative*	*Higher*	My child was sad/upset/frightened &	52	Level 1	6	11.5
	∣∣	My child was fussy/cranky/irritable &		Level 2	7	13.5
	∣∣	My child was tired/sleepy/hungry		Level 3	24	46.2
	∣∣					
	∣∣					
	*Lower*			Level 4	15	28.8

*Level represents the arousal level of music—Level 1 being most arousing and stimulative and Level 4 being most relaxing and sedative. Level 1: Playlists 3 & 7; Level 2: Playlists 1, 5, & 8; Level 3: Playlists 2 & 4; Level 4: Playlists 6 & 9.*

#### Modulating Music to Promote Well-Being

Mothers also reported any perceived effects that music might have had on their children while playing the role of home DJ. Overall, music appeared to help children stay positive and improve mood (81.2%) although there were cases when mothers did not notice any effects or reported that playing recorded music actually deteriorated their children’s mood (13.7%). Positive effects of music listening were observed particularly in episodes where children were in a positive mood and exhibited a relatively high activity level. Following our suggestion, mothers played music more often with an expectation to maintain the positive mood (89 episodes). In more than half of these episodes, children actively reacted to music by singing along, moving their bodies to the beat, and dancing (50.6%). A mother commented after playing Playlist 1 for her two toddlers (ages 2 and 3):

It was snack time, and the kids were very lively and happy… When music was on, they immediately responded to the music. They stood up and started to hop on the chair…, moving their head to the beat.

When highly arousing music (i.e., Level 1) was used, some reports reflected active parent-child and sibling musical interactions taking place in the home environment. This is well-depicted in a comment of a mother of two toddlers (ages 1 and 3).

… when the first song [*Banana Chacha* in Playlist 7] began, my older one immediately started dancing and asked me to join, and then we all had a little dance party. She kept requesting to play back the first song, which I did, so we listened to this song more than 10 times, dancing and having lots of fun together.

In addition, some episodes (12.4%) suggested that music eased children’s daily routines and transitions. A mother of two active boys (ages 1 and 4), described how the arousing and stimulative music playlist (i.e., Playlist 7) helped her 4-year-old son burn off energy after repeating it twice: “The music seemed to reinforce his hyper-activeness, which knocked him down by the nap time.” Another mother of a 2-year-old boy, who described her son as a “picky eater” in an earlier comment, mentioned that music helped her son have a pleasant meal time: “I played it[Playlist 1] while he was having lunch. He enjoyed the music, moving his body side by side along with the music. Then he happily finished his lunch, which I loved.”

The positive effects of music were also commonly observed when children were in a positive mood, but relatively low in their activity level (56 episodes). In these episodes, implicit listening was frequently observed (59%), as mothers intentionally put on the music in the background while their children were engaged in other activities. Many mothers reported that implicit listening helped children stay focused on their own activities for a longer time, regardless if children were conscious about the music or not. A mother of a 4-year-old put on Playlist 4 while her daughter was engaging in a daily worksheet activity and later commented:

I’m not sure if it was due to the music, but today, with the music in the background, she focused on her worksheet like 10 min longer than her usual attention span. Also, she didn’t say things like “I don’t wanna do this” or “I can’t do it” that she would normally do and happily finished her work.

While this study only consisted of mother participants, a few comments suggested that their spouses learned to use music to maintain their children’s positive mood as well. A mother shared an incident that her husband, who did not know much about the study, put on music for their 2-year-old son with a particular expectation in mind:

My younger one woke up early in the morning so my husband took care of him since I needed more sleep. When I woke up after a while, I went to the living room and saw my son playing with toys and with this playlist[Playlist 1] in the background. It was my husband who played it from my YouTube library and [he] told me that my son plays by his own better when we have this music on. It was interesting to me because I haven’t particularly mentioned this study to my husband but he somehow knew [this playlist]–BTW, he works from home so he often sees my kids throughout the days.

Also, some episodes (10.9%) illustrated how music was effective to invigorate children when they were drowsy or having hard time to wake up in the morning or after a nap. Mothers reported that children turned their attention to and became reactive to the music, which eventually helped them get into a good mood. A mother of a 2-year-old girl, who played Playlist 1 on her way to drop off her half-asleep child to her parent’s house for babysitting in the morning, commented:

From the first song, she paid attention to the music [and gradually]… got into the rhythm with her head moving along with it. She enjoyed most of the tracks… She smiled and became lively and active. And definitely she was fully awake with a smile by the end of the playlist.

Interestingly, the situations where mothers found the least effects of music were when children were in uncomfortable states physically and/or mentally (52 episodes). Over 40% of these episodes revealed that music did not help or even worsen their children’s mood. A mother of a 4-year-old boy shared a comment after she attempted to use music to help her child improve his mood:

He didn’t feel well since we woke up in the morning. I played this one[Playlist 2] with a hope that it can be helpful to make him feel better. Disappointedly it didn’t and he got even worse as he noticed that the music wasn’t something he wanted.

Nonetheless, mothers reported various positive experiences after playing the role of home DJ over a period of one week. When they were asked to share their overall experiences at the end of the week, many mothers expressed an appreciation for the opportunity to learn how to use music to modulate their children’s mood. A mother of two toddler girls commented:

“Your study has introduced me to a new way of keeping my second child (2 yr old) to stay calm and restful with your playlist, especially Peaceful Minds[Playlist6]. Also a good study to help me, as a parent, to manage stress since it had elevated my mood as I play the [play]lists and observe my children responding to them.”

As implied in her comment, children’s positive mood in turn aided to improve mothers’ mood and thus boost their overall psychological well-being. A comment by a mother of 2- and 4-year-old children articulated this aspect, “As a mom of two toddlers, their sanity is at priority since it directly influences my well-being. Music works like magic.”

Even on occasions where music did not directly affect their children, it helped some mothers relax and de-stress, as the playlists were designed for both children and adults to enjoy. This is particularly valuable because recent studies on parenting during the time of Covid-19 pandemic suggested parents’ stress to be associated with parent-child closeness and parenting styles (Chung et al., 2020) and child’s well-being (Spinelli et al., 2020). In addition, and as discussed earlier, several mothers’ comments highlighted the function of music to help with daily routines and transitions. Keeping with daily routines, such as mealtime, naptime, and bathtime, is not only important for mothers with young children, but is related to the well-being of the parents and the family (Spagnola and Fiese, 2007). These episodes implied that incorporating music into daily routines and transitions also helped, to some extent, to reduce some of the burdens associated with parenting.

Lastly, as mothers actively played the home DJ role over the week, they discovered strategic ways to use music with their children while also expanding their family music listening repertoire. Such comments were found from mothers who reported having a very limited music repertoire (i.e., children’s music to be an exclusive listening repertoire for their child), and from those who reported a relatively low frequency of music use with their children in the initial survey. A mother of a 2-year-old boy, who was not used to incorporate various musical styles/genres into their daily listening before taking part in the study, commented about how the introduction of new music through the playlists helped her to broaden their family listening repertoires, “I didn’t know that my son would enjoy this kind of music, as I had only played children’s music on YouTube for him. I’m very glad that I now have music that both of us can enjoy together.” Similarly, another mother of two toddler girls commented:

I usually don’t play music for my kids often. That’s just my personal style, I guess. I started to use music a lot as I participated in this study, and realized that music really made me and my kids stay positive and happy. My younger one doesn’t cry much anymore as I started to put some music on every morning.

## Discussion and Conclusion

As the outbreak of COVID-19 has significantly limited outdoor activities and increased time in the home, this marked great shifts in the everyday lives of young children and their families. This exploratory study was designed to examine parents’ experiences of utilizing recorded music for their children in the home environment over a period of one week called “Parents as Home DJs week” using a modified version of ESM that we developed. Given the amount of parental burnout that has been documented since the emergence of COVID-19, we anticipated that playing the role of a home DJ would lead participants to discover some new parenting ‘tricks’ that likely help both their children and parents stay happier and less distressed during this challenging time. Overall, 19 participating mothers’ reports over the week showed that as they paid careful attention to their children’s mood and emotional states while playing the home DJ role, they found various effects of music on routine maintenance and children’s well-being. Detailed findings emerged from the responses of all 19 mothers to the initial survey and the 197 episodes from the initial surveys are discussed here.

First and foremost, the survey data showed that mothers utilized recorded music to fulfill various emotional needs in their everyday lives. Consistent with previous studies (Trehub and Schellenberg, 1995; Ilari, 2005; Trehub et al., 2010), children’s mood and states were often used as indicators of when and what kinds of music mothers put on. Overall, mothers tended to use music to maintain or reinforce their child’s positive mood rather than to improve a negative mood, given that out of the 197 episodes, only 26% were related to situations when children were in uncomfortable states physically and/or mentally. This seems somewhat different from how adults engage in music listening. Previous literature showed that while adolescents and adults listened to music most often for emotional reasons (Schäfer et al., 2013; Schäfer, 2016), the most popular emotional reason was to regulate their affective experiences when they were in a negative mood, for example, to relax/de-stress, improve mood, cope with a situation, vent emotion, and forget problems (Randall and Rickard, 2017). Interestingly, using music to alter mood often failed to elicit changes in the valence and arousal when adult listeners were in a negative mood and experiencing low arousal (Randall and Rickard, 2017). This partially echoes the mothers’ report, as they found the least effects of music when dealing with their child’s negative affect. Even if the reason that music is less effective in alleviating negative mood is unclear, the selected repertoire may have a role in this. Given that the playlists recommended for soothing and calming effect tended to be low-arousing, sedative, and, to some extent, “sad” pieces, it is possible that listening to such music resulted in an intensification of children’s adverse emotional states, making them more irritated and tired (Randall et al., 2014; Carlson et al., 2015). Further investigation is needed to see if relatively more arousing, stimulating, and happy music functions better in regulating children’s negative mood.

Another notable finding relates to the various ways that young children engaged in music (Ilari, in press). The most frequently observed listening mode was implicit where music played in the background, accompanying non-musical activities, such as drawing, playing with toys, and having meals/snacks. When mothers chose to put on some music to help their child stay positive for a longer duration, many episodes indicated that music effectively achieved that goal, which reflects the close link between “passive” music listening and emotional well-being found for adult populations (Västfjäll et al., 2012; Randall et al., 2014). In such cases, children did not seem to be aware of the music, showing no overt reaction to it, yet their focus on the non-musical activities tended to extend. Reactive listening was also frequently observed, particularly when mothers selected highly arousing music in response to children’s positive mood and high activity level. Children reacted to music by moving to the beat, singing along, and dancing to the music, which helped to reinforce their positive mood. Musical interactions between mothers and children and between siblings were often observed with this reactive mode of listening. The self-initiated mode appeared in two distinct situations: when children liked particular music and when they disliked their mother’s music selection. In the latter case, children insisted on replacing it with their favorite music and this tended to happen more often when children were in a negative mood and/or physically tired. It should be noted that although children’s responses to their mother’s music selection were often reported, we rarely found episodes where the child initiated his or her own music listening, which may highlight the significance of the caregiver’s role to craft the sonic environment for young children. The deliberate mode was the only one that was not observed in our data. Given that we introduced some musical activities that parents could do with their children using the playlists through IG posts, we expected to see at least some episodes related to deliberate mode but no one actually reported those. Although this could imply that mothers preferred to use music during free play with no explicit educational purpose at home, it is possible that they may simply have not considered the activities much since we did not place any emphasis on using music for intentional learning activities.

Children’s listening repertoire is also worthy of discussion. Many mothers in this study seemed to hold their own criteria in terms of what counted as “children’s music.” There was a trend suggesting that mothers’ personal listening repertoire was not compatible with what they offered to their children. This trend was particularly distinct in popular music genres, including pop/rock/hip-hop, R&B/Soul and jazz, but mothers who liked to listen to classical and religious music tended to share these repertoires with their children. Nonetheless, a few mothers still appeared to listen to their favorite pop songs with their children, with a mother reporting them as her child’s favorite songs. Survey data further suggested that mothers’ personal criteria of “children’s music” limited their choices of repertoires that were presented to children. As the playlists included a variety of musical genres, including different styles of popular music, some mothers reported that they recognized much wider ranges of music that is “okay” to share with their children after participating in the current study. Taken together, we assume that, although no consensus existed on what music was “appropriate” for young children (Ilari, 2005), mothers in our study held their own perceptions about it, directly influencing their child’s early musical experiences. In addition, it was clear that the multimodal nature of musical experiences was also apparent in children’s listening repertoires, particularly as mothers reported children’s listening to and preferences for “Pinkfong,” “Super Simple Songs,” and other musical videos available on online video platforms like YouTube.

Fourth, it is vital to comment on the associations between music listening, maternal satisfaction with the parental role and perceived well-being that emerged from our data. While cause and effect cannot be inferred here, it is possible that shared music listening experiences induced positive emotions, fostered parent-child bonding and generated well-being, resulting in higher levels of satisfaction with parenting. This explanation is consistent with earlier research on parent-child bonding in and through music (Custodero and Johnson-Green, 2003; Trehub et al., 2010), the power of music to induce positive emotions (Yehuda, 2011), and musical engagement and well-being (Croom, 2012). Future studies could further determine whether music listening frequency can predict satisfaction with parenting and parental self-efficacy. This is important because parental satisfaction and well-being trickle down to children and families (Prime et al., 2020). As seen in this and many other reports and popular press stories, the pandemic has exacerbated the ‘crisis of care’, with mothers shouldering additional childcare obligations (Fodor et al., 2020), which directly impacts their well-being. As a potent, non-pharmacological mood-inducer, music could be used as a means for relaxation, entertainment, attachment and well-being in families. As our data showed, armed with simple daily reminders and curated playlists, parents may find ways to bond with their children during these times of stress and reclusion.

Finally, it may be necessary to comment on the sample of this study. Although this study was open to any primary caregivers of young children in the United States, the study participants were Asian-American mothers. Most were highly educated, with half of them holding arts-related or education-related jobs. It is highly possible that these characteristics may have affected how these mothers utilized recorded music with their children. Yet, we did not find evidence that mothers’ education, occupation, and prior music training experiences had an impact on the patterns of their music use. This is not conclusive, however, given that we only collected limited information about participants’ background. Also, consistent with many previous parenting research studies (e.g., Williamson et al., 2017; Mingebach et al., 2018), all participants were mothers. Although we cannot ascertain if the study findings will hold if fathers were to play a home DJ role, we anticipate that this role can be played by any adult caregivers. Overall, data from the initial survey suggested that the participating mothers actively played recorded music for children in their everyday lives even before they took part in the Parents as Home DJs week. Previous research on Asian-American parents (e.g., Hsin and Xie, 2014; Kim, 2021) highlighted a great interest in child’s cognitive development compared to parents from other ethnic groups. Asian-American parents are also known for their active role in shaping the home environment from the early years of life. Thus, mothers’ active uses of music with children found in this study may reflect an implicit expectation of music to facilitate extra-musical outcomes. Earlier studies on musical parenting of Asian (Youm, 2013; Cho, 2015) and Asian-American mothers (Hwang and Cho, 2019) also suggested that they value music as a powerful means to support emotional stability and contentment in childhood, which may also explain their active incorporation of music in daily lives. Finally, it is also possible that our findings reflect mothers’ compliance to the directives of our study. But if this were the case, we would have seen similar numbers of reports on uses of music to assist a distressed child and to maintain positive mood. Future study should investigate this question and also examine if parents’ ethnic background plays a role in the ways they use music with young children and reasons to incorporate it in their daily lives.

As the second aim of our study, we also attempted to test a newly developed data collection tool that incorporated various digital technologies. As the circumstances surrounding the COVID-19 pandemic have challenged researchers to conduct experimental research, we developed a data collection tool, inspired by the ESM. It not only enabled us to conduct the study without face-to-face contact with participants, but it was also particularly effective with this specific study population, parents with young children, whose time availability tends to be inconsistent and inflexible. IG, the main platform of the study, allowed us to asynchronously communicate with the participants yet maintaining close access to each other. Whenever necessary, we reached out to the entire participants through posts, likes, and comments, and also to individual participants via the DM, and they were able to check them out at their convenience throughout the day. By posting various musical parenting resources and study-related information on the IG account multiple times each day, we could indirectly encourage participants’ active engagement in the study throughout the week. Given that our goal was to lead participants to actively play the home DJ role and report their experiences daily, IG was particularly effective in achieving this goal while avoiding giving them unnecessary pressure. In addition, IG was effective to comply with privacy and confidentiality regulations associated with experimental research–while some participants set their accounts as private, others chose to create a new account for their participation in the study. Therefore, we had no access to their personal information other than what they had shared through the surveys.

As is the case with any data collection tool, this one also had some limitations. Given participants’ anonymity in the virtual setting, we had little control over their engagement in the study during the week. Although we initially had 21 mothers who agreed to participate and filled out the initial survey, 19 of them actually completed all tasks. Among them, four mothers filled out less than 4 daily surveys. Other than sending various forms of encouragement through IG, there was no other way to motivate the participants without being a nuisance. This suggests that when conducting a research study that involves multiple tasks over time in the virtual setting using digital platforms, careful and deliberate planning should be made to increase social presence, or the feeling of being present with a “real” person and having access to the partner’s thoughts and emotions in virtual contexts (Biocca et al., 2003), to sustain participants’ motivation for active engagement for the duration of the study. Using visual representations of the researchers (i.e., video + audio formats) may help to increase participants’ sense of social presence (Oh et al., 2018). While we solely relied on IG in communicating with the participants, we incorporated various infographics and texts, but no audio or video representation was utilized. Given that social presence is closely linked to trust, enjoyment, and persuasion (Oh et al., 2018), the absence of visual representation of ourselves as communication partners may have lowered some participants’ perceptions of social presence, which possibly resulted in their low engagement. Additionally, since we intended to keep the daily survey as simple as possible to improve answering efficiency, mothers with multiple children were unable to report episodes for each child separately. Although their comments in the open-ended comment helped us to understand which child each episode referred to, this prevented us from performing further analysis on the effects that music had on individual children. Despite some potential drawbacks, this newly developed tool enabled us to collect data on young children and their mothers’ real-life engagement with music listening in the home during the COVID-19 pandemic.

Although more investigation is needed, findings from our study shed some light on the potential power of music listening to promote well-being in children and families when parents strategically craft the sonic home environment. The pandemic has not only increased the quantity of time that parents are spending with their children, but has also forced them to rethink the quality of the time spent together. As the pandemic lingers on, so does the need to examine what strategies may maximize the effects of and potential benefits of music listening in young children and their families, during this challenging and stressful time.

## Data Availability Statement

The raw data supporting the conclusions of this article will be made available by the authors, without undue reservation.

## Ethics Statement

The studies involving human participants were reviewed and approved by University of Southern California. Written informed consent for participation was not required for this study in accordance with the national legislation and the institutional requirements.

## Author Contributions

EC and BI contributed to study design, data analysis, and manuscript preparation. Both authors contributed to the article and approved the submitted version.

## Conflict of Interest

The authors declare that the research was conducted in the absence of any commercial or financial relationships that could be construed as a potential conflict of interest.
